# Circulating progenitor cells and the expression of *Cxcl12*, *Cxcr4* and *angiopoietin-like 4* during wound healing in the murine ear

**DOI:** 10.1371/journal.pone.0222462

**Published:** 2019-09-12

**Authors:** Clare E. Yellowley, Chrisoula A. Toupadakis, Natalia Vapniarsky, Alice Wong

**Affiliations:** 1 Department of Anatomy, Physiology and Cell Biology, School of Veterinary Medicine, University of California Davis, Davis, California, United States of America; 2 Department of Pathology, Microbiology and Immunology, School of Veterinary Medicine, University of California Davis, Davis, California, United States of America; Ludwig-Maximilians-Universitat Munchen, GERMANY

## Abstract

Migration of cells from both local and systemic sources is essential for the inflammatory and regenerative processes that occur during normal wound healing. CXCL12 is considered a critical regulator of CXCR4-positive cell migration during tissue regeneration. In this study, we investigated the expression of *Cxcl12* and *Cxcr4* during healing of a murine full thickness ear wound. We also investigated the expression of angiopoietin-like 4, which has been shown to participate in wound angiogenesis and reepithelialization. At time points up to 48hrs, complete blood counts were performed using automated hematology analysis, and the numbers of circulating stem and progenitor cells quantified using flow cytometry. Expression of both *Cxcr4* and *Angptl4* was significantly elevated within 3 days of wounding, and both were strongly expressed in cells of the epidermis. ANGPTL4 protein expression remained elevated in the epithelium through day 14. *Cxcl12* expression was increased significantly at day 3, and remained elevated through day 21. Faint *Cxcl12* staining was detectable in the epithelium at day 1, and thereafter staining was faint and more generalized. There were significantly fewer circulating total white blood cells and lymphocytes 1hr following ear punching. Similarly, there was a significant early (1hr) reduction in the number of circulating endothelial progenitor cells. Further studies are warranted to investigate whether ANGPTL4 and CXCL12/CXCR4 interact or synergize to facilitate cell recruitment and migration, and to potentiate reepithelialization and wound healing.

## Introduction

Tissue healing requires the mobilization, recruitment, migration and homing of cells to the site of damage. Cells recruited during the initial inflammatory response include neutrophils and macrophages, the migration of which is facilitated by a variety of chemotactic signals including those released by damaged cells, activated platelets, and tissue resident immune cells among others [1]. Endothelial and mesenchymal stem and progenitor cells are recruited to take part in tissue regeneration along with more committed/differentiated cells of these lineages. Such cells can be derived from local sources such as the epidermis, dermis, blood vessel walls and hair follicles [1]. However, stem and progenitor cells may also be recruited from the peripheral circulation. Few circulating stem and progenitor cells can be detected under normal conditions. However, the numbers of circulating stem and progenitor cells are significantly elevated in response to injury, burns and fracture [2–9]. All of these cells play a specific role in the tissue healing cascade but in order to do so must navigate through local tissue and home to the site of damage. This migration is orchestrated by the release of potent chemotactic agents at the site.

Chemokines are small, 8-14kDa chemotactic cytokines responsible for the establishment of chemical gradients for cell migration. There are four subfamilies, CXC, CC, (X)C and CX3C based on the position of the N-terminal two cysteine residues and their receptors are G-protein coupled receptors, classified into the same four subfamilies [10]. CXCL12 is a CXC family member and binds to chemokine (CXC motif) receptor type 4 (CXCR4)[11] to promote the chemotactic recruitment of stem and progenitor cells. Indeed, CXCL12/CXCR4 signaling is thought to be a master regulator of stem cell migration [12]. CXCL12 expression has been shown to increase rapidly at sites of ischemic damage in tissues such as bone, heart and brain [13–15].

It is possible that chemokines are not solely responsible for stem and progenitor homing and migration subsequent to injury, but work in tandem with other locally sourced molecules. For example, high gene expression of angiopoietin-like 4 has been demonstrated in the murine epithelium shortly after the generation of a full thickness skin wound [16] and we have shown that angiopoietin-like 4 mRNA (*Angptl4*) levels are increased during regeneration of bone after fracture [17]. Angiopoietin-like 4 is an adipocytokine that has been shown to play a role in keratinocyte differentiation [18] and migration [16], wound angiogenesis [19] and reepithelialization [20]. In this study, we utilized a through and through ear defect model in the mouse to investigate mobilization of endogenous stem and progenitor cells into the peripheral circulation in the early stages of wound healing. We expand on previous studies by simultaneously examining endothelial progenitor cell (EPC), hematopoietic stem cell (HSC) and mesenchymal stem cell (MSC) populations in the blood. We also evaluated the expression of *Cxcl12* and *Cxcr4* in the wound. In addition, we determined the expression of angiopoietin-like 4 mRNA and protein at the wound site.

## Materials and methods

### Ear wound model

All procedures were approved by the Institutional Animal Care and Use Committee of the University of California, Davis (Protocol number 17423). 13-week old C57/BL6 mice were anesthetized under 1.5–2% isoflurane, and all efforts were made to minimize suffering. While under anesthesia, the right ear was cleaned with 70% ethanol and a 2mm circular wound was created using a 2mm diameter sterile biopsy punch (Integra Miltex, Plainsboro, NJ, USA). To determine ear wound area, some mice were placed on a light table under anesthesia. The ears were taped to the light table surface using clear tape and photographed with a digital camera outfitted with a macro lens (Pentax K2000 Digital SLR, Ricoh, Tokyo, Japan). Ear wounds were photographed weekly for 8 weeks under anesthesia until euthanasia. For time-point day 0, wound area was calculated using the diameter of the 2mm biopsy punch. For all other time-points, wound area was calculated from digital images using ImageJ (National Institute of Health). Animals used for histology and in situ hybridization were euthanized at days 0, 1, 2 and 3; for quantitative real time PCR at 0, 1 and 6hrs and days 1, 2, 3, 5, 7, 14 and 21; for immunohistochemistry at days 0, 1, 2, 3, 7 and 14, and for peripheral blood analysis at time points up to 48hrs.

### Peripheral blood collection, hematological analysis and flow cytometry

Peripheral blood (500–1000μL) was collected from anesthetized mice by cardiac puncture into 100μL of 50mmol/L ethylenediamine tetra-acetic acid (EDTA) at time points up to 48hrs following ear punching. Complete blood counts were performed on 50μL aliquots of peripheral blood using an Advia 120 hematology analyzer (Siemans Healthcare Diagnostics, Deerfield IL, USA). For flow cytometry, the remaining peripheral blood was treated with ammonium chloride (Life Technologies) on ice for 15min to lyse red blood cells and stained with Live/Dead Fixable Near Infrared Viability Kit (Life Technologies). After incubation at room temperature for 20min, 5μL of fetal bovine serum (Life Technologies) was added to bind any remaining dye. The following antibodies were added as a cocktail and incubated at room temperature for 20min in the dark; Ter119 (APC-Cy7), B220 (APC-C7), CD3 (APC-Cy7), Gr-1 (APC-Cy7), c-kit (Brilliant Violet 421), CD135 (PE-Cy5), CD29 (Alexa Fluor 700) and CD44 (Brilliant Violet 570), from Biolegend (San Diego, CA, USA); CD150 (PerCp-eFluor 710), from eBioscience (San Diego, CA, USA); Flk-1 (PE-Cy7), from BD Pharmingen (San Jose, CA, USA); CD105 (PE), from eBioscience; CD34 (FITC), from BD Biosciences (San Jose, CA, USA); sca-1 (APC), from eBioscience. Flow cytometry data was acquired as described [21] using a LSRII flow cytometer (Becton Dickinson, Franklin Lakes, NJ, USA) and analyzed with the use of FlowJo software (Treestar, Inc, Ashland, OR, USA). A summary of flow cytometry markers for each stem and progenitor cell population is shown in Table 1.

**Table 1:** Flow cytometry markers.

Cell Type	Positive Markers	Negative Markers	References
Hematopoietic stem and progenitor cell (HSPC)	c-kit, sca-1	B220, CD3, Ter119, Gr-1	[22]
Hematopoietic stem cell (HSC)	c-kit, sca-1, CD150	B220, CD3, Ter119, Gr-1, CD135	[23]
Mesenchymal stem/stromal cell (MSC)	Sca-1, CD29, CD105	CD31, CD34, CD44, B220, CD3, Ter119, Gr-1	[24,25]
Endothelial progenitor cell (EPC)	FLK-1, CD31, CD34	B220, CD3, Ter119, Gr-1, CD44, CD29, CD150, CD135	[26–28]

### Histology, *in situ* hybridization and immunohistochemistry

After euthanasia, ears were removed, fixed in 4% phosphate-buffered formalin for a minimum of 48hrs at 4°C, paraffin-embedded and sectioned (4μm thick). Sections taken from an area that bisected the circular wound were deparaffinized, rehydrated and stained with hematoxylin and eosin. For *in situ* hybridization, slides were probed with *Angptl4*, *Cxcl12 and Cxcr4* RNA using the following primers *Angptl4*; F 5’-ccagactcctgagactctgc-3', R 5’-gcacagccaattggcttcc-3’, *Cxcl12*; F-5’-gtcctcttgctgtccagctc-3’, R-5’-taatttcgggtcaatgcaca-3’, and *Cxcr4*; F-5’-ttctcatcctggccttcatc-3’, R-5’-atggagttgagtgcatgctg-3’. A T7 sequence was included at the start of all reverse primers generated using T7 transcription kit (Roche, Indianapolis IN). Colorimetric visualization was performed using AP substrate-chromogen NBT/BCIP (Sigma). Negative controls were generated by eliminating incubation with the probe. For immunohistochemistry slides were blocked with Background Buster (Innovex Biosciences), and stained with anti-ANGPTL4 (ThermoFisher 40–9800, 1:20). Visualization for immunohistochemistry was achieved using Stat-Q AEC kit (Innovex Biosciences). Negative controls were generated by elimination of incubation with the primary antibody. All slides were evaluated by a board-certified veterinary pathologist, blinded to the groups.

### Quantitative PCR analysis

After euthanasia, the ear was removed and a circular piece of tissue was taken using a 6mm biopsy punch; the center of the wound was used to center the punch and the resultant tissue sample was “donut” shaped, the inner edge of which was the wound margin. For zero hour time points a 2mm defect was created in an intact ear and a 6mm biopsy punch used as described above to collect a tissue sample. RNA was isolated using TRIzol (Invitrogen, Carlsbad, CA) and cDNA was generated using the QuantiTect Reverse Transcription kit (Qiagen, Valenica, CA). Quantitative reverse transcription PCR (qPCR) was performed using primer and TaqMan probe sets (LifeTechnologies) and QuantiFast Probe PCR kit (Qiagen) on a Mastercycler realplex2 (Eppendorf, Westbury, NY). PCR products were amplified under the following conditions: 95°C for 3 min, followed by 40 cycles at 95°C for 3s and 60°C for 30s. The following genes were amplified: angiopoietin like-4 (*Angptl4*, Mm00480431_m1), collagen Type 1 alpha 1 (*Col1a1*, Mm00801666_g1), Cxcl12 (*Cxcl12*, Mm00445553_m1), and Cxcr4 (*Cxcr4*, Mm01996749_s1). Quantitative PCR results were normalized to beta-actin (*ActB*, Mm00607939_s1) to yield ΔC_t_.

### Statistics

Data are presented as means and error bars represent standard error of the mean. Statistical analysis of means was performed using one-way ANOVA with Tukey’s multiple comparisons test. A probability level of p < 0.05 was used to consider differences statistically significant.

## Results

### Gross and microscopic analysis of murine ear wound healing

**Fig 1:**
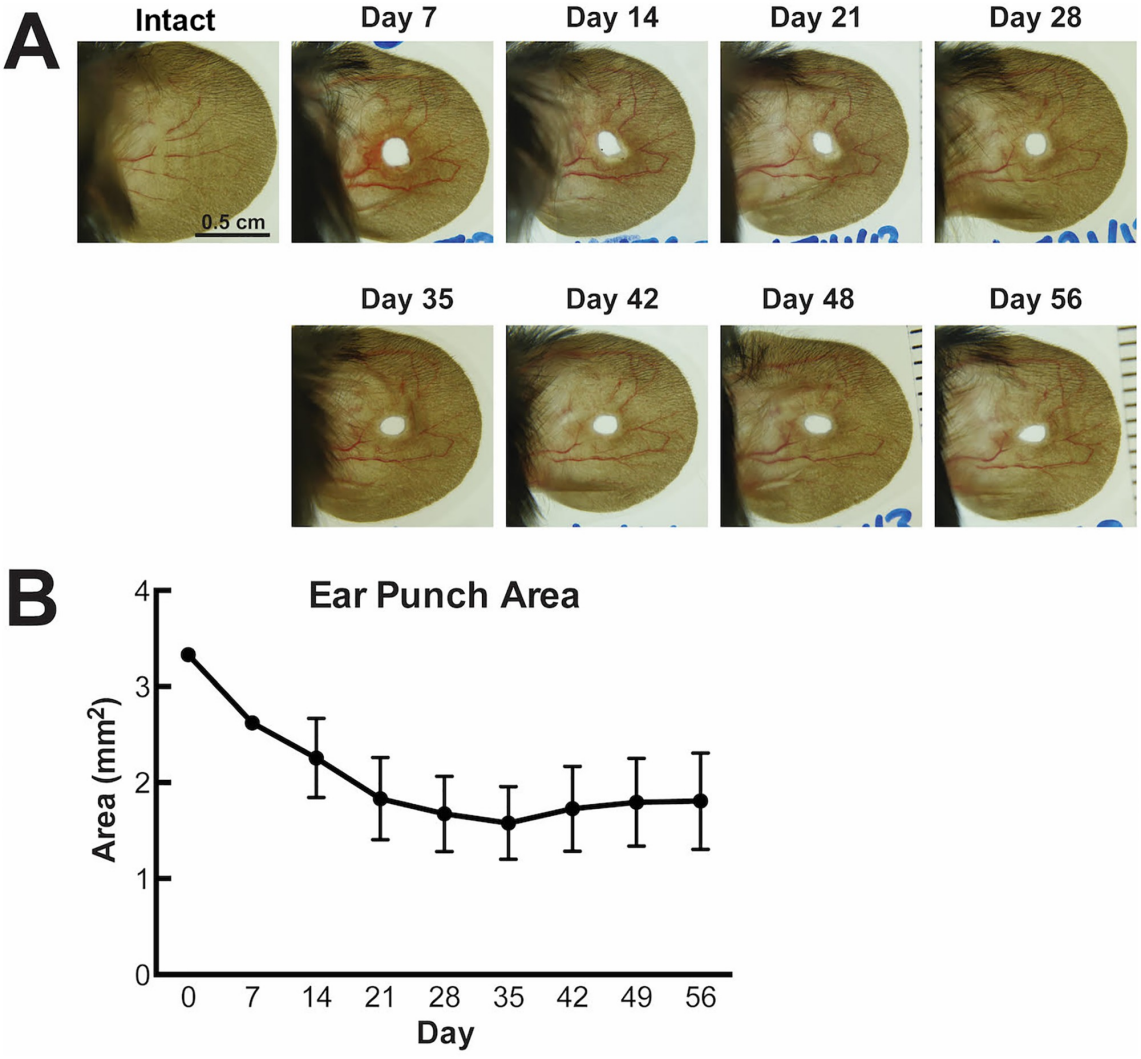
Normal wound healing of a 2mm critical size defect in the murine ear. A) representative photographs of the murine ear prior to wounding (intact), and of healing at time points up to 8 weeks after a punch wound. B) Wound area over time. Each point represents the mean wound area (mm^2^) ± SEM, n = 6.

The ear punch produced a full thickness, 2mm diameter wound in an area where the ear is supported by a core of elastic cartilage. Fig 1A shows the reduction in wound diameter over time and demonstrates that the wound heals without full closure. Closure of around 55% was achieved at 4 weeks, after which the wound diameter plateaued, Fig 1B.

**Fig 2:**
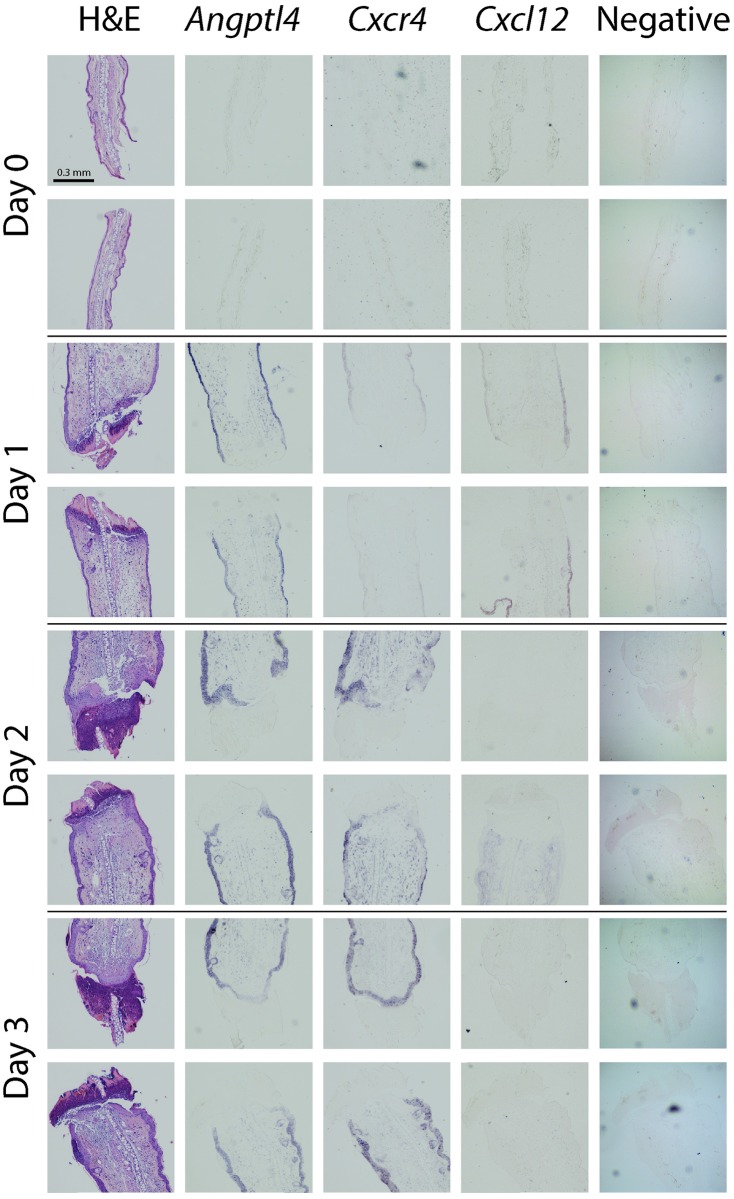
Histology of the ear wound and detection of *Angptl4*, *Cxcr4*, *and Cxcl12 mRNA*. Hematoxylin and eosin staining (H&E) and detection of *Angptl4*, *Cxcr4*, *and Cxcl12* RNA hybridization in sequential cross-sections through ear wounds at 0, 1, 2, and 3 days after wounding. All images were taken at 10x magnification. First column: H&E stains of both sides of the ear wound. Second-fourth columns: corresponding *in situ* hybridization staining of *Angptl4*, *Cxcr4*, *and Cxcl12*, respectively. Scale bars (0.3mm) are indicated on top-left panel only, however the scale is uniform throughout all panels. Images are representative of n = 3.

On day 0, the wound margins exhibited no overt histologic changes, Fig 2.

On day 1 the wound margins were covered by 20μm thick sero-cellular crust, Fig 2. The cellular component of the crust was comprised primarily of degenerate neutrophils. Similarly, the margins of the viable dermis were infiltrated by predominantly neutrophils, histiocytes and occasional lymphocytes. A thin layer of granular amphophilic material separated the sero-cellular crust and viable dermis (fibrin). Interestingly the cartilaginous core of the pinna was minimally affected and remained in a proud position relative to the wound surface, surrounded by afore mentioned sero-cellular crust. In the viable dermis at the wound margins, the small terminal lymphatic channels were dilated. There was mild perivascular edema and minimal lymphocytic infiltration. The keratinocytes at the margins of the wounded epidermis were slightly piled up and hypertrophied.

On day 2 the thickness of the sero-cellular crust increased to 30 μm and the cellular component remained predominantly neutrophilic, Fig 2. The layer of granular amphophilic material (fibrin) separated the wound crust and subjacent viable dermis, and was infiltrated by moderate numbers of viable and degenerate phagocytic cells such as histiocytes and neutrophils. The viable cartilage core was in sunken position relative to the surface of the wound, while previously proud in position. The viable dermis was infiltrated by lymphocytes with fewer neutrophils and histiocytes. The epidermal margins were thickened and curved towards the center of the wound in the direction of the cartilaginous core. There was prominent hypertrophy and piling up of the marginal keratinocytes.

On day 3, the thickness of sero-cellular crust increased to 40μm, with neutrophils remaining the predominant cell type, Fig 2. The wound margins were completely covered by epidermis (reepithelialization) subtended by previously described fibrin substance. This substance contained multiple large spindle cells admixed with rare neutrophils and histiocytes. The cartilage core remained in sunken position and showed no evidence of degeneration. The viable dermis still contained a moderate number of infiltrating lymphocytes, fewer neutrophils, histiocytes, and occasional mast cells. Clear spaces previously occupied by edema fluid, were replaced by granular basophilic matrix (granulation tissue).

### *In situ* hybridization, quantitative PCR analysis and immunohistochemistry

#### Angiopoietin-like 4

**Fig 3:**
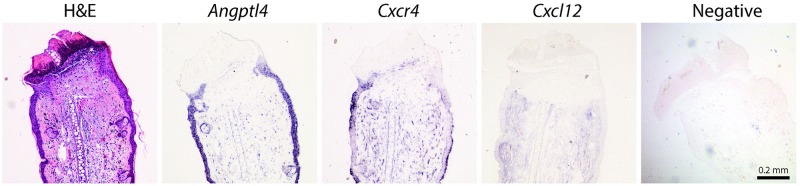
Histology of ear wound at day 2. High power images of H&E, and RNA staining for *Angptl4*, *Cxcr4*, *and Cxcl12* at day 2. Images are representative of n = 3.

**Fig 4:**
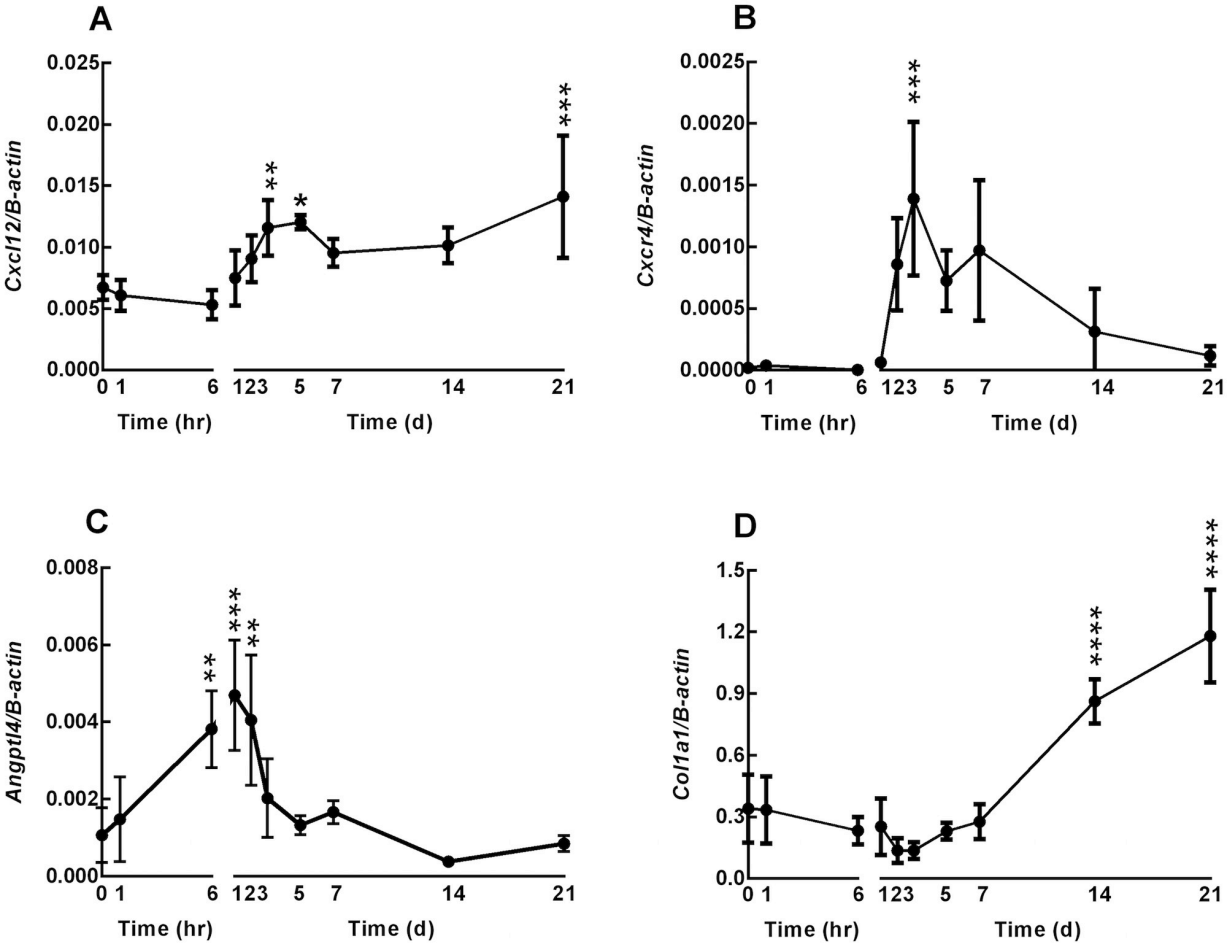
Gene expression during wound healing. A. *Cxcl12*, B. *Cxcr4*, C. *Angptl4* and D. *Col1a1*. Expression levels were measured at 0, 1 and 6 hr and from 1–21 days following ear punch. Results are expressed as mean ΔCT ±SEM, normalized to beta-actin transcript level, n = 3–6. Data significant by one-way ANOVA to time zero as indicated (*p<0.05, **p<0.01, ***p<0.001, ****p<0.0001).

**Fig 5:**
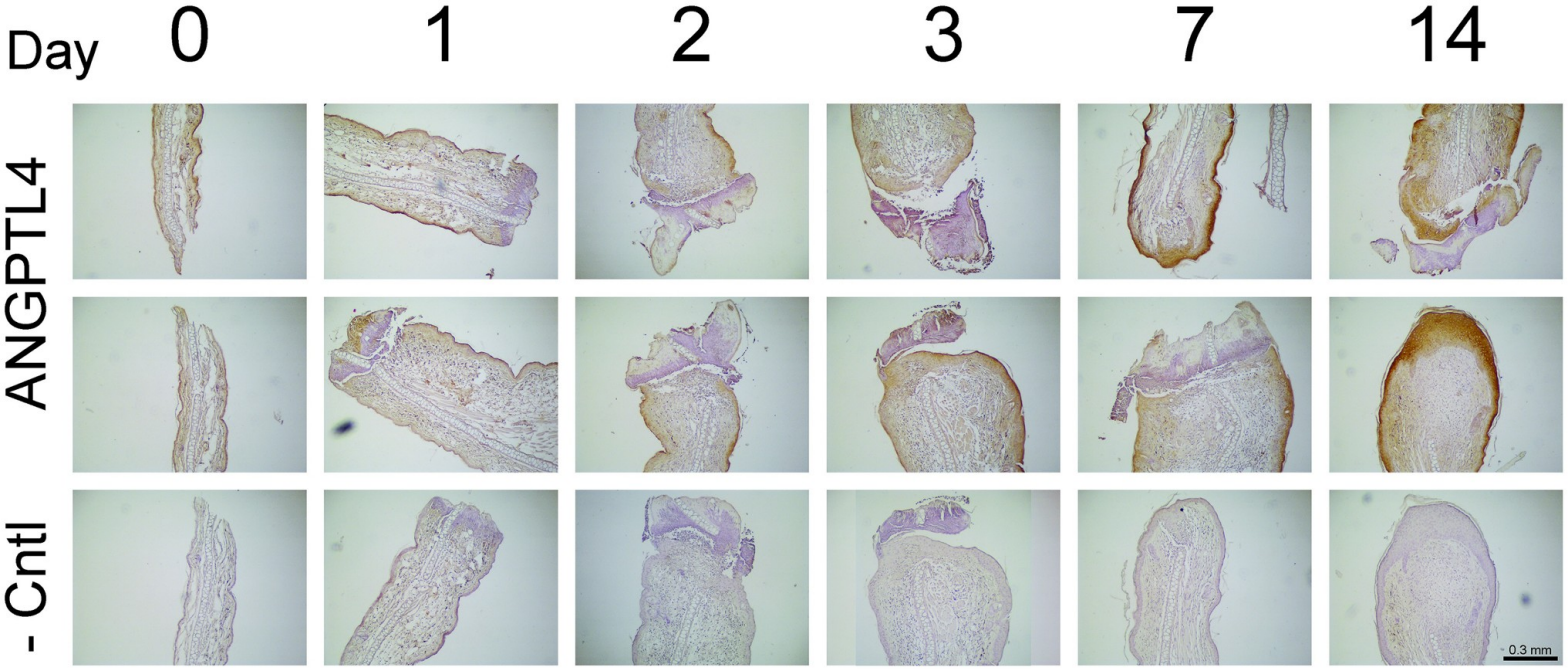
Immunohistochemical staining of ANGPTL4. ANGPTL4 staining (brown) at the wound site immediately (day0) after wounding, and at days 1, 2, 3, 7 and 14 after wounding. Images are representative of n = 3.

There was little detectable *Angptl4* mRNA at the wound site immediately (day0) after wounding, Fig 2. However, 1 day after wounding, there was strong staining in the epithelium, Fig 2. A number of cells with fibroblastic morphology stained positive in the underlying loose connective tissue and no staining was evident at the healing edge of the wound at day 1. At days 2 and 3 strong staining was maintained in the epithelium, Figs 2 and 3. The regenerating epithelium close to the wound margins stained positive, ultimately covering the whole wound site at day 3. Of note, cells at the base of the hair follicles and sebaceous glands stained positive for *Angptl4*, Fig 3. *Angptl4* mRNA expression increased rapidly following ear wounding and was significantly increased over baseline at 6hr, 1 and 2 days, Fig 4C. Maximal expression levels were seen at day 1 after which levels declined to near baseline at day 5, Fig 4C. There was visible but light staining of ANGPTL4 in the epithelium at the wound site immediately (day0) after wounding, Fig 5. From day 1–7 there was a progressive increase in ANGPTL4 staining in the epithelium. At day 14 strong staining was visible; at the edge of the wound where the new epithelium was clearly evident, and the crust had fallen off, there was particularly strong staining of the epithelial cells, Fig 5.

#### Cxcl12

*Cxcl12* mRNA levels increased significantly at day 3 and remained elevated through day 21, Fig 4A. Using ISH, faint *Cxcl12* staining was detectable in the epithelium at day 1, Fig 2. Thereafter staining was faint and more generalized, Figs 2 and 3.

#### Cxcr4

*Cxcr4* mRNA expression increased rapidly between day 1 and day 2 reaching maximum expression levels at day 3, Fig 4B. Expression declined thereafter, approaching baseline levels at day 21. The pattern of *Cxcr4* expression detected by ISH was similar to that detected by QPCR. There was little detectable *Cxcr4* mRNA detected at the wound site immediately (day 0) after wounding, and 1 day later there was faint staining in the epithelium, Fig 2. However, at days 2 and 3 strong staining was apparent in the epithelium and the pattern of staining closely mirrored that of *Angptl4*, including positive staining of hair follicles and sebaceous glands, Figs 2 and 3.

#### Col1a1

*Col1a1* expression was significantly increased at days 14 and 21, Fig 4.

### Hematology analysis and flow cytometry

**Fig 6:**
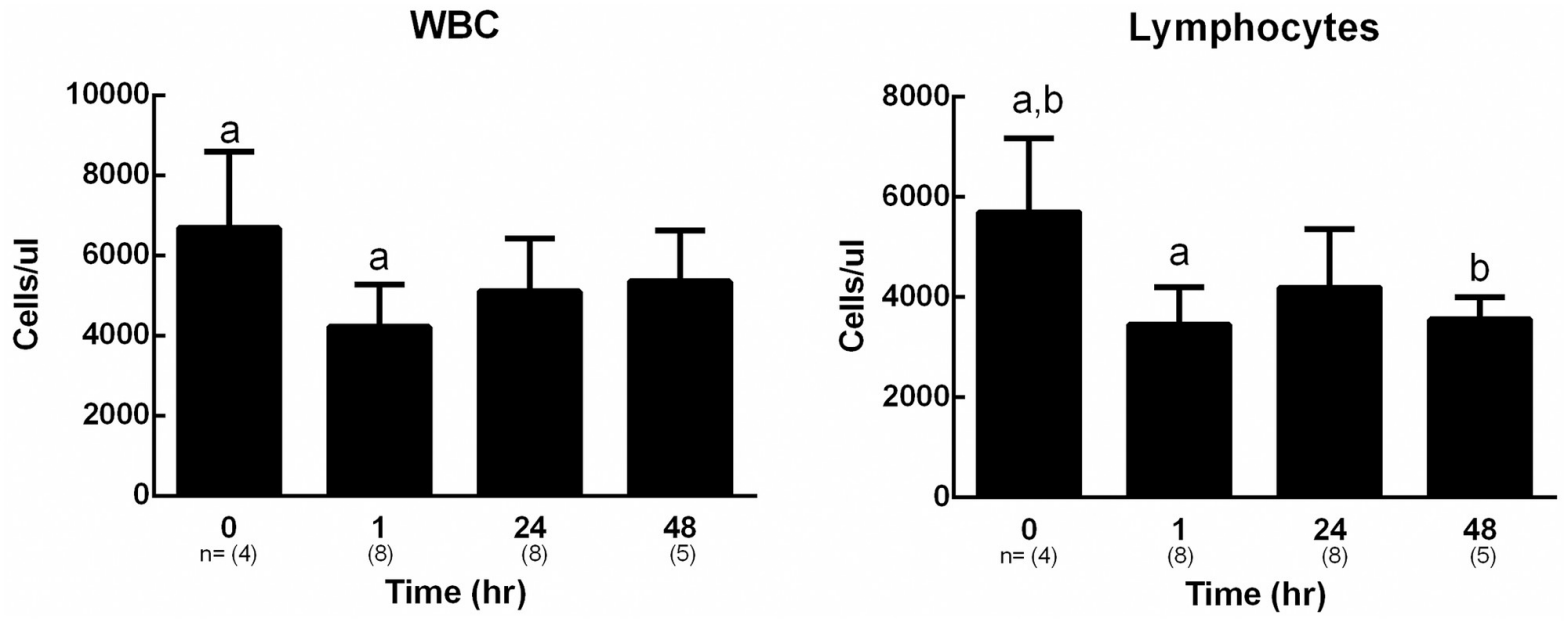
Circulating white blood cell and lymphocyte numbers. White blood cell and lymphocyte counts (Advia 120) from wounded mice at time points between 0 and 48hrs. Bars represent mean ±SEM, n = 4–8 (indicated in brackets below each bar). Bars with the same letters are significantly different from one another, p <0.05.

Complete blood counts indicated no significant changes in the numbers of circulating neutrophils, basophils and monocytes up to 48hrs following the ear punch (supplemental data S1 Fig). However, there were significantly fewer circulating total white blood cells and lymphocytes 1hr following ear punching, Fig 6.

**Fig 7:**
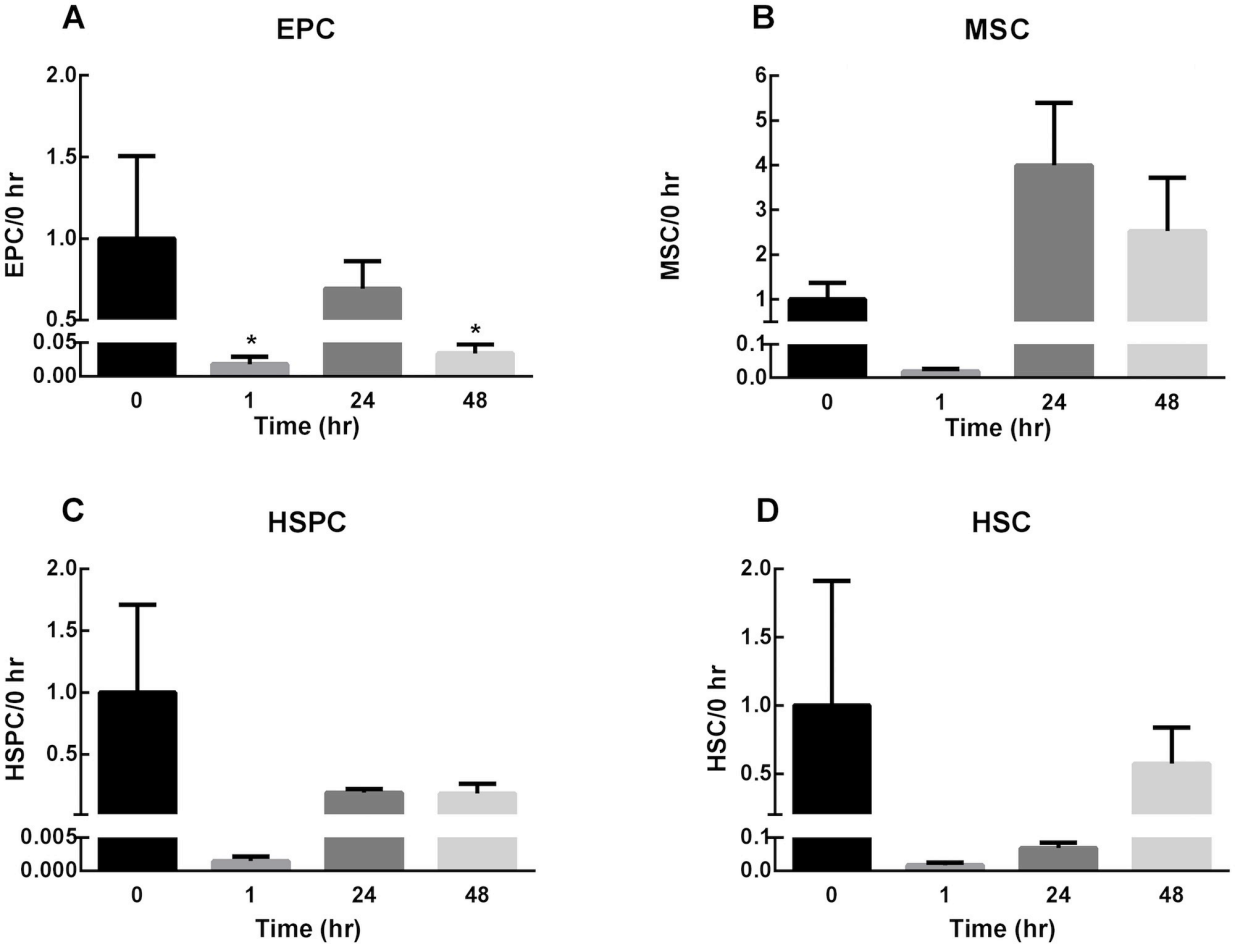
Circulating progenitor cell numbers. Flow cytometry counts of EPCs, MSCs, HSPCs, and HSCs (as defined in Table 1), reported in fold change versus time 0. Blood was collected from mice immediately (0hr), 1, 24 and 48hrs after ear wounding. Bars represent mean fold change ±SEM, n = 3. * represents p<0.05.

We defined progenitor cell subsets according to the markers outlined in Table 1. The number of circulating EPCs was significantly reduced 1hr following ear punch, Fig 7A. Circulating numbers rebounded close to original levels at 24hrs and were significantly reduced once again at 48hrs, Fig 7A. There was a trend for HSPC, HSC and MSC numbers to decrease at 1hr but this did not reach statistical significance, Fig 7B, 7C and 7D.

## Discussion

*Angptl4* mRNA was strongly expressed in the first 3 days of wound healing, with expression levels particularly high in the epithelium and wound bed. These findings are similar to those of Goh and colleagues 2010 [16], who demonstrated high expression of *Angptl4* in the epithelium at day 3, in a murine full thickness skin wound. Similar to Goh and colleagues 2010 [16], protein levels remained elevated in the epithelium long after *Angptl4* levels had returned to baseline. Wound reepithelialization has been shown to be impaired in *Angptl4* knockout mice, but could be rescued by topical application of ANGPTL4 [16]. Diabetic (ob/ob) mice demonstrate delayed wound healing and reduced *Angptl4* expression; topical application of ANGPTL4 accelerated reepithelialization in these mice also [19]. *Angptl4* deficient keratinocytes have impaired migration [16] and differentiation capacity [18]. *Angptl4* knockout mice showed impaired epidermal differentiation with increased numbers of apoptotic cells and decreased proliferative cells at the wound site [18]. ANGPTL4 has been shown to interact with integrin β1 and β5 to enhance integrin mediated signaling and keratinocyte migration [16]. In addition to effects on keratinocytes, recombinant ANGPTL4 influenced wound angiogenesis, increasing CD31 expression and the number of CD31+ endothelial cells in skin wounds of diabetic mice [19]. Interestingly, tendon fibroblasts from *Angptl4* knockout mice exhibited delayed migration, while recombinant ANGPTL4 increased fibroblast proliferation [29]. *Angptl4* was also increased during healing of bone fractures [17] which suggests a critical role for ANGPTL4 in the healing cascade in multiple tissues. How ANGPTL4 may influence the migration, homing, and differentiation of circulating and local stem and progenitor cells, and whether it interacts with the CXCR4/CXCL12 axis remains to be investigated.

It was noted that cells at the base of the hair follicles and sebaceous glands stained positive for *Angptl4*. Interestingly, in a human immortalized sebaceous gland (SG) cell line induced to undergo sebaceous lipogenesis, ANGPTL4 levels progressively increased as sebaceous lipogenesis advanced [30]. Depletion of ANGPTL4 with siRNA in SG cells undergoing lipogenesis, increased the number of lipid droplets, decreased their mean area and increased the total cellular lipid content[30]. However, there were no changes in SG size, and no changes in the composition and amount of hair lipids in *Angptl4*-/- mice [30]. It was concluded that although ANGPTL4 appears to regulate sebaceous lipogenesis, the lack of effect seen in *Angptl4*-/- mice was most likely due to as yet unknown upregulation of compensatory mechanisms.

CXCL12 expression in normal human skin has been shown to be largely restricted to the dermis while CXCR4 was predominantly expressed in the epidermis [31]. Specifically, CXCL12 was detected in normal human, swine, mouse and rat skin, in the basal layer of the epidermis and in various cell types in the dermis including fibroblasts, pericytes, endothelial layer of blood vessels, hair follicles, sweat glands and nerves [32,33]. *In vitro*, CXCL12 was expressed in dermal fibroblasts but not in keratinocytes; in contrast, keratinocytes but not fibroblasts expressed CXCR4 [31,33]. In response to wounding, we report increased expression of *Cxcl12* which remained elevated through day 21, and a significant increase in *Cxcr4* expression that was transient in nature, peaking at day 3. There was striking expression of *Cxcr4* in the epidermis, but more diffuse staining of *Cxcl12* in the epidermis and dermis. This is consistent with previous studies that showed that *Cxcl12* levels were significantly increased in the skin following burn injury or skin wounding [32–35]. In response to burn injury, CXCL12 was upregulated in human burn blister fluid, the basal layer of the epidermis, and in fibroblasts, endothelial cells and hair follicles of the recovering dermis [33,36]. In wounded rat skin, *Cxcl12* was present in the basal layer of the epidermis, scattered cells in the dermis and was increased through day 7, and *Cxcr4* was highly expressed in the epidermis [34]. In a murine skin wound, CXCL12 was strongly expressed in epidermal keratinocytes, fibroblasts and endothelial cells [32]. AMD3100 which disrupts CXCL12/CXCR4 signaling, inhibited CXCL12 stimulated migration of epidermal stem cells *in vitro* [34]. In addition, CXCL12 accelerated wound closure in rats while AMD3100 delayed closure [34]. There was an increase in epidermal cell numbers in rats treated with CXCL12, suggesting that the positive effects of CXCL12 on healing were via stimulation of epidermal cell proliferation and migration.

In addition to stimulating local cell migration, studies also suggest that CXCL12/CXCR4 signaling facilitates wound healing by recruiting progenitor cells to the wound site to aid in repair. In mice that received a transplant of bone marrow derived-MSCs from GFP transgenic mice, a skin burn injury resulted in recruitment of GFP positive MSCs to the wound margin at day 1 [36]. Cells were localized to the epidermis and hair follicles [36]. BM-MSC migration and wound closure were inhibited by AMD3100 [36]. DiI-labeled BMSCs infused into mice with skin wounds, accumulated at the wound site and promoted wound closure [32]. Inhibiting CXCR4/CXCL12 signaling with either anti-CXCR4 or anti-CXCL12 antibodies decreased BMSC migration and delayed healing [32]. The numbers of circulating stem and progenitor cells have been shown to be significantly elevated in response to injury, burns and fracture [2–9]. Specifically, EPC numbers were shown to be elevated within 2 days of traumatic bone fracture in human patients [37]. In contrast, in this study we showed that the numbers of circulating white blood cells decreased significantly 1 hour post-wounding, possibly due to a significant decrease in circulating lymphocytes, and demonstrated a significant decrease in the number of circulating EPCs. In our previous studies we have shown that in mice, AMD3100 effectively mobilized white blood cells into the peripheral circulation, but that circulating numbers were significantly lowered in the presence of a fracture [38]. We theorized that the presence of an injury caused the cells to rapidly marginate and migrate to the wound, effectively reducing circulating cell numbers. Injected progenitor cells have been identified in the lung and bone defects as early as 24 hours [39] indicating how rapid this process might be.

In summary, we have demonstrated early upregulation in expression of *Cxcl12*, *Cxcr4*, and Angiopoietin-like 4 mRNA and protein in skin wounds. There was striking upregulation of both *Angptl4*, ANGPTL4 and *Cxcr4* in the epidermis. In addition, we show a significant decrease in circulating white blood cells and EPCs following wounding. Further studies are warranted to investigate whether ANGPTL4 and CXCL12/CXCR4 interact or synergize to potentiate reepithelialization and wound healing, and whether this involves effects on local cell migration and differentiation and recruitment of circulating cell populations.

## Supporting information

S1 Fig Circulating neutrophil, basophil and monocyte cell numbers. Neutrophil, basophil and monocyte counts (Advia 120) from wounded mice at time points between 0 and 48hrs. Bars represent mean ±SEM, n = 2–8 indicated in brackets below each bar.
(TIF)
