# Supplementary figures and images for: Circulating progenitor cells and the expression of Cxcl12, Cxcr4 and angiopoietin-like 4 during wound healing in the murine ear
Source: PLoS One. 2019 Sep 12;14(9):e0222462. doi: 10.1371/journal.pone.0222462 (PMC6742462; doi:10.1371/journal.pone.0222462)

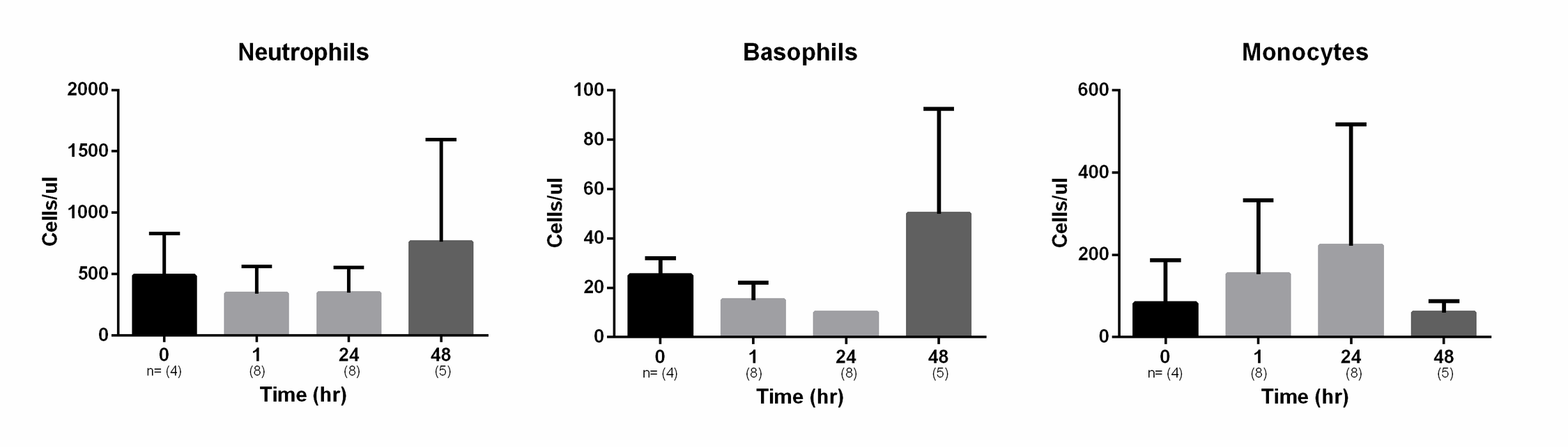

Supplement: S1 Fig — Neutrophil, basophil and monocyte counts (Advia 120) from wounded mice at time points between 0 and 48hrs. Bars represent mean ±SEM, n = 2–8 indicated in brackets below each bar. (TIF) [file pone.0222462.s001.tif]
